# Monitoring the transition to new antiretroviral treatment regimens through an enhanced data system in Kenya

**DOI:** 10.1371/journal.pone.0232104

**Published:** 2020-04-23

**Authors:** Maria Lahuerta, Maureen Syowai, Shobha Vakil, Jacob Odhiambo, Mwenda Gitonga, Nandita Sugandhi, Stanslaus Odhiambo, Maureen Kimani, Joyce Wamicwe, James Batuka, Evans Imbuki, Kigen Bartilol, Elaine J. Abrams

**Affiliations:** 1 ICAP at Columbia University, Mailman School of Public Health, New York, NY, United States of America; 2 Department of Epidemiology, Mailman School of Public Health, Columbia University, New York, NY, United States of America; 3 Palladium, Nairobi, Kenya; 4 National AIDS & STI Control Programme (NASCOP), Nairobi, Kenya; 5 United States Agency for International Development (USAID) Kenya, Nairobi, Kenya; 6 Department of Pediatrics, Vagelos College of Physicians & Surgeons, Columbia University, New York, NY, United States of America; NPMS-HHC CIC / LSH&TM, UNITED KINGDOM

## Abstract

**Background:**

While the scale-up of HIV services has improved national health management information systems (HMIS), there remain challenges in using routine data to guide the introduction of optimized antiretroviral (ARV) drugs.

**Methods:**

Building on the recent enhancements to the HMIS in Kenya and coinciding with the introduction of a new ARV regimen, tenofovir+lamivudine+dolutegravir (TLD), we developed and implemented an enhanced data system (EDS) to improve availability of safety and efficacy data among people living with HIV (PLHIV) in Kenya. Using data from one health facility, we showcase how the EDS can be used to monitor ARV transition and identify missed opportunities to transition eligible patients to optimized regimes.

**Results:**

The EDS was designed to create a comprehensive PLHIV database by triangulating patient-level data from the EMR, the pharmacy ARV dispensing tool (ADT) and HIV viral load (VL) databases. On a monthly basis, the database is de-identified and uploaded into a national data warehouse, with interactive dashboards. Using the EDS, we determined that of the 5,500 PLHIV ≥15 years on first-line ART at one facility, 4,233 (77%) had transitioned to optimized ARVs. Of the 1,267 still on legacy regimens, 459 (36%) were determined to be eligible and prioritized to switch.

**Conclusions:**

This project illustrates how enhancements to the national HMIS can facilitate the use of routine patient-level data to monitor the transition to new ARVs and inform the national HIV response.

## Introduction

The scale-up of HIV prevention and treatment services in sub-Saharan Africa has provided a unique opportunity to improve national health management information systems (HMIS) [1, 2]. Many countries have started to transition from paper-based to electronic medical records (EMR) to document information including medical history, visit information, medications and laboratory studies. Additionally, increasing coverage of internet connectivity in many settings has enabled the implementation of online electronic systems, making it easier to obtain and use patient-level data at the national level for program monitoring and planning. Kenya has been a leader in this arena, making extraordinary enhancements to the national HMIS. For example, the National AIDS & STI Control Program (NASCOP) has implemented an integrated national data warehouse of EMRs from health facilities providing HIV services. The warehouse includes interactive dashboards to enable users to visualize key indicators from the EMR aggregated at the national level. As of February 2019, 918 facilities were submitting monthly data from their EMR systems to the national data warehouse, capturing 37% of people living with HIV (PLHIV) receiving HIV services in Kenya.

Recent advances in HIV antiretroviral (ARV) drug development hold the promise of delivering more efficacious, safe and well-tolerated treatment options for PLHIV [3, 4]. In 2017, NASCOP introduced an optimized antiretroviral treatment (ART) regimen, tenofovir (TDF) + lamivudine (3TC)+dolutegravir (DTG) [TLD], which also presented an opportunity to document the safety and efficacy of TLD outside a research setting. Despite the advances in the HMIS in Kenya, there were still some limitations to using routine data to monitor ARV safety and efficacy on a national level [5]. First, the existing pharmacovigilance system to document ARV-related adverse events (AE) was passive; i.e, patients were not actively monitored for AEs at each clinical encounter and there was no standard approach to documenting AEs within the EMR. Additionally, the national laboratory viral load (VL) database was not triangulated with the EMR. Viral load results were downloaded in the laboratory from the national VL website and entered individually into the EMR, resulting in long delays, errors and high rates of missing data. Finally, the pharmacy ARV dispensing tool (ADT) database tracks ARV drugs and regimens dispensed, while the EMR captured information on the ARV regimen prescribed to the patient. The data between the ADT and EMR were not triangulated because of different patient identifiers in each database. This created inconsistencies between the two systems in the total number of people currently on different regimens resulting in discrepancies in the number of PLHIV reported on ART at the national level.

Building on the recent enhancements to the HMIS in Kenya and coinciding with the introduction of TLD, we developed and implemented an enhanced data system (EDS) to triangulate data from different electronic systems and improve availability of safety and efficacy outcomes among PLHIV in selected health facilities in Kenya. In this manuscript we describe the EDS and present an example of how this system is being used to monitor the transition to optimized ARV regimens.

## Materials and methods

### Setting

Twenty-four public health facilities in Kenya were purposively selected by NASCOP for initial transition to TLD in 2017. Of these, four health facilities were prioritized for development and implementation of the EDS in 2018, including Jaramogi Oginga Odinga Teaching and Referral Hospital (JOOTRH), Siaya County Referral Hospital, Machakos Level 5 Hospital and Nyeri County Referral Hospital.

### Transition to optimized ARV regimens in Kenya

In 2017, Kenya became the second country in sub-Saharan Africa, and the first in East Africa to introduce TLD in its national ART program. [6] Since 2014 the preferred first-line regimen had been TDF/3TC/efavirenz (TLE), but many PLHIV had continued on other legacy regimens such as zidovudine(AZT)+lamivudine(3TC)+nevirapine(NVP). The current guidelines recommend transition to TLD, after confirmation of viral suppression (VS), defined as VL <1000 copies/μL, measured within the past six months, for adolescents (≥15 years) and adult men. TLE is recommended for women and adolescent girls (≥15 years) of childbearing potential. Women and adolescent girls who are on effective contraception may opt to use TLD. [6].

### Data sources

The NASCOP HMIS captures key PLHIV outcomes through different electronic and paper-based data systems at the facility and national level. The electronic systems include clinical information systems from the national EMR, the pharmacy ADT and the national laboratory VL database.

The EMR documents all information collected at clinical visits, including socio-demographic information, clinical assessments, ARV regimens and clinical outcomes including co-morbidities and pregnancy status. As such, the EMR serves as the primary source of information regarding baseline and longitudinal clinical data for PLHIV. The EDS has been designed using the KenyaEMR, IQCare and E-care EMRs currently implemented in Kenya.

The pharmacy ADT database (Web ADT) tracks ARV drugs and regimens dispensed, reasons for drug substitution, number of days/months of drugs dispensed and pharmacy appointments. The data on the ADT database is usually recorded by a pharmacist or pharmacy technologist.

Finally, the online VL database is a national laboratory database that serves as the primary source for all VL test results [7].

### Adverse events documentation

**Fig 1:**
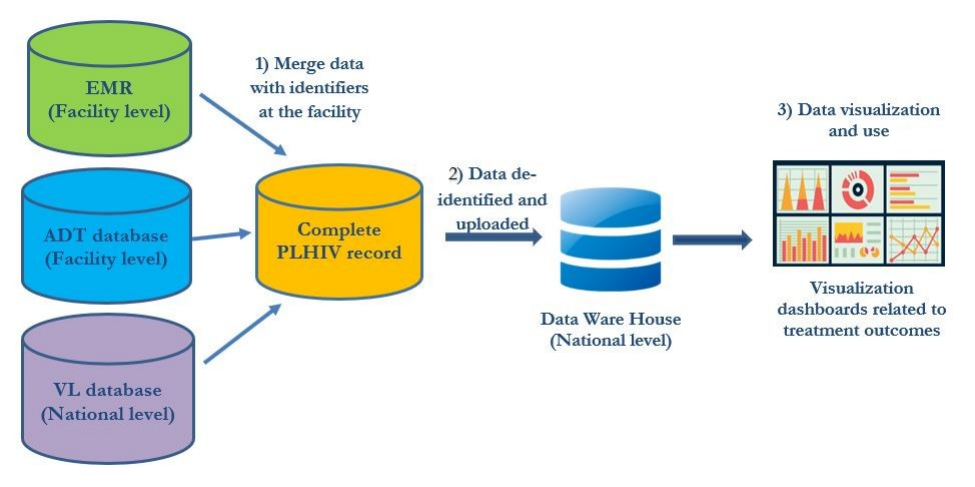
Data flow of the enhanced data system to monitor safety and efficacy of new optimized ARVs.

An AE screening form was developed in order to institute an active approach to toxicity monitoring among PLHIV and ensure HCW review and report on the presence or absence of AE at each clinical encounter (Fig 1). The form documents patient reported AE, causality and actions taken including drug substitution, ARV regimen changes and treatment discontinuation. The form also captures AE resolution, hospitalization and death. This AE screening form was integrated into the EMR so that data could be captured electronically to allow for active routine monitoring and reporting of adverse drug events.

### Ethical considerations

The use of data from the EDS was approved by the Scientific and Ethics Review Unit at the Kenya Medical Research Institute (KEMRI) and the Institutional Review Board at Columbia University Medical Center. The requirement of consent was waived by these institutions given that the data was fully anonymized before being used for analysis.

## Results

### Description of the enhanced system

The data flow for the EDS is presented in Fig 1. An interoperability layer was created to link clinical data from the EMRs, including AE data, with ART dispensing and VL results from the ADT and VL databases, respectively. Given the lack of unique patient identifiers, a master person index (MPI) was developed to link the individual records across all three systems. The MPI uses pre-determined common patient identifiers, including clinic identification number, first and last name and date of birth. The interoperability layer merges data from the three different databases into a complete PLHIV record, providing a comprehensive source for providers to access patient information in one unique location.

On a monthly basis, data in the merged database are de-identified and uploaded to the national data warehouse, where built-in dashboards are automatically updated using Tableau software. For example, one dashboard includes graphs with the number and type of AE. The EDS allows stakeholders to monitor utilization of different regimens, uptake of new optimized ARVs and AEs of patients on specific ARVs and ART regimens by sub-population (e.g. by sex or age).

All data is de-identified at the facility level. Patient privacy and system security procedures were developed following the national and international standards for EMR and health informatics [8, 9].

### Data from the enhanced system

At the four facilities where the EDS has been implemented, there were 13,261 active ART patients by the end of February 2019, of which 615 (5%) were children <15 years old. Of the 12,646 adults ≥15 years, 8,383 (66%) were women including 2,126 (25%) on TLD (Table 1). Similarly, there were 4,262 (34%) adult men on ART, of whom 1,773 (42%) were on a TLD.

**Table 1:** Antiretroviral regimen among active people living with HIV, by sex and age, February 2019, at four health facilities, Kenya.

	Overall	Adults (≥15 years old)						Children (<15 years)	
		Women			Men				
		Total	TLD-regimen		Total	TLD-regimen		Girls	Boys
Facility*	N	N	n	%	N	n	%		
JOOTRH	6222	3934	1008	26%	1988	1048	53%	166	134
Siaya	3557	2120	343	16%	1222	416	34%	111	104
Machakos	1052	721	221	31%	320	99	31%	7	4
Nyeri	2430	1608	554	34%	732	210	29%	39	51
**TOTAL**	**13,261**	**8,383**	**2,126**	**25%**	**4,262**	**1,773**	**42%**	**323**	**293**

Jaramogi Oginga Odinga Teaching and Referral Hospital (JOOTRH), Siaya County Referral Hospital, Machakos Level 5 Hospital and Nyeri County Referral Hospital; TLD: tenofovir+lamivudine+dolutegravir

### Using the EDS to monitor the transition to TLD at JOOTRH

In February 2019 NASCOP convened a team of experts to review the transition to TLD. Using the EDS data, they conducted an assessment on ARV optimization in JOOTRH using a Rapid Response Initiative (RRI) approach. We present the findings from this review to illustrate how the EDS can be used to monitor the transition to TLD.

**Fig 2:**
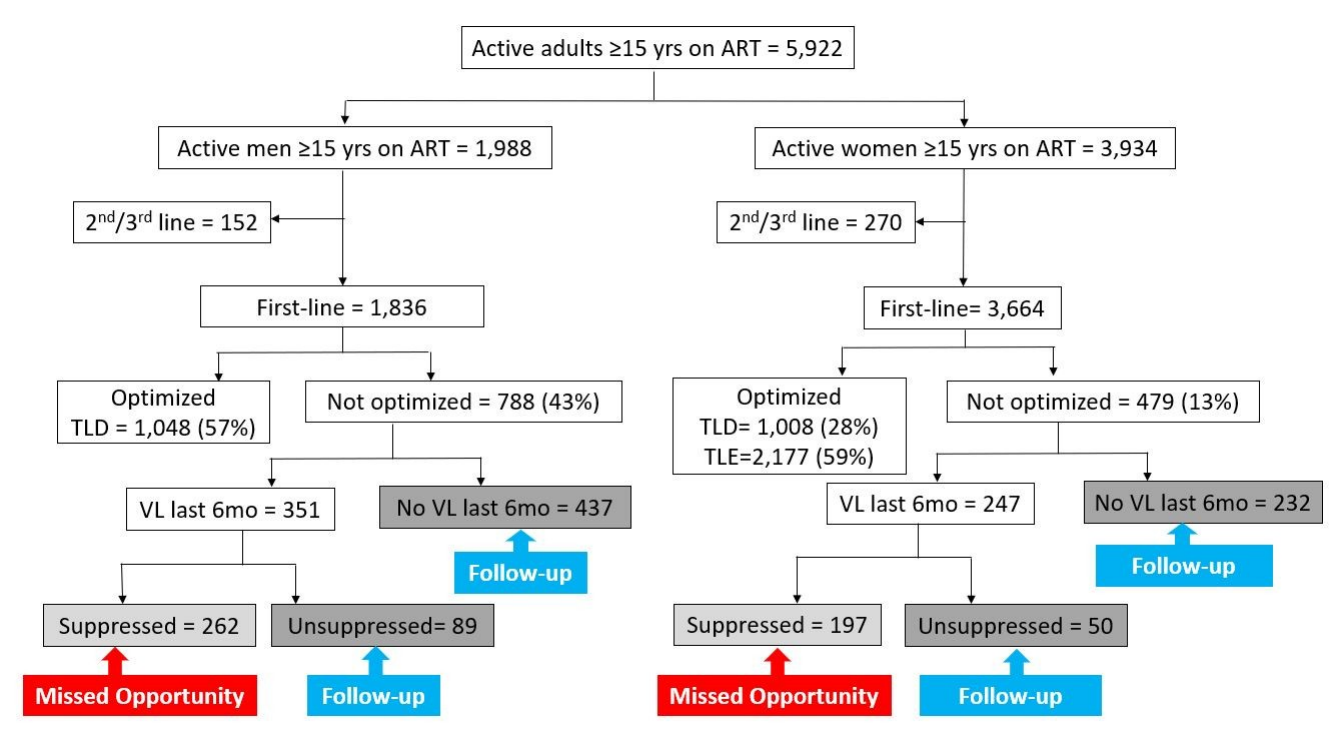
Example of the use of the enhanced data system to monitor antiretroviral drug optimization transition, by sex, at Jaramogi Oginga Odinga Teaching and Referral Hospital (JOOTRH), Kisumu, Kenya, February 2019. ART: antiretroviral treatment; JOOTRH: Jaramogi Oginga Odinga Teaching and Referral Hospital; TLD: tenofovir+lamivudine+dolutegravir; TLE: tenofovir+lamivudine+efavirenz; VL: Viral load.

By February 2019, there were 6,222 active PLHIV on ART at JOOTRH, of which 5,922 were adults ≥15 years (Fig 2). Out of the 5,500 PLHIV (1,836 men and 3,664 women) on first-line regimen, 4,233 (77%) had transitioned to optimized ARVs. Of these 1,048 men and 1,008 women (28%) were on TLD (Fig 2). There were also 2,177 women (59%) on TLE, at the time of review, still the first-line recommended for women of childbearing potential not using effective contraception.

Overall 1,267 PLHIV (788 men and 479 women) were not on the optimized first-line ARV regimen, 598 PLHIV (351 men and 247 women) (47%) had a VL test done in the last six months and 459 PLHIV (262 men and 197 women) were VS. Overall, 459/1267 (36%) were eligible to transition to an optimized regimen and were thus prioritized by clinicians for switch. The majority were targeted for additional follow-up including viral load testing for those with no recent test [669 PLHIV (437 men and 232 women)(52%)] or enhanced adherence counseling [139 PLHIV (89 men and 50 women)(11%)] for those with elevated VL.

## Discussion

This project illustrates how enhancements to the national HMIS can facilitate the use of routine patient-level data at the national level to monitor the transition to new ARVs as well as to inform the national HIV response. We also present an example of how the data from the EDS can be used at the facility level to optimize patient care and to monitor transition to optimized ARV. The facility data presented in this manuscript highlight the progress made to date at rapidly transitioning PLHIV to optimized ARVs in Kenya, a country that has been at the forefront of global optimization efforts. The EDS also provided the national program and the facility health workers with a simplified and direct approach to identify patients eligible for VL testing, ARV optimization or enhanced adherence counseling.

While the EDS has only been implemented to date at four facilities, the next step is to continue implementation of the EDS to 20 additional facilities that have been prioritized by NASCOP. At these 20 facilities, the AE screening form has already been integrated in the EMR, and healthcare workers have already been trained on AE screening, management and reporting of identified AEs using this form. This has transformed the collection of safety data from a passive to active process. Once the EDS is implemented at a health facility, HCW will be able to obtain a complete PLHIV record from the EMR, the pharmacy and the VL database, with complete VL data and accurate information on ARVs dispensed.

While the use of real-life routine data in this project is one of the strengths of this system, allowing for closer monitoring and program improvements, this can also be a limitation, since routinely collected service data often has missing or incorrect information compared to data collected as part of a research study. In low and middle-income countries, the quality of routine data tends to be sub optimal, as health workers are often overburdened and do not have the time nor appropriate training to document the required information accurately [10]. To mitigate this, we conducted additional training and mentoring and implemented data quality assurance activities to improve data quality.

The EDS offers clear enhancements from previous systems, but it also underscores the complexities in developing interoperable data systems, given the extensive back-end programming needed to make the datasets interoperable and to de-duplicate patients without a unique identifier. NASCOP is currently implementing additional enhancements to make this a fully operational system. One of this enhancement includes the development of dashboards, in the national data warehouse, that would allow providers and managers at all levels of the health system to use the EDS data to easily stratify ARV uptake and AE and VS by age, sex or regimen. Given the availability of different EMRs in Kenya, additional efforts will also be needed to make all the EMRs interoperable with the VL and ADT databases. Finally, the recent implementation of a national unique identification number in Kenya will minimize problems of interoperability across systems and facilitate the tracking of individuals across facilities.

Overall, our project highlights how routine patient-level data can be used to provide safety and efficacy data at the national level to inform scale-up of optimized ARVs. In order to maximize the benefits of more effective ARV regimens, there is an urgent need to develop innovative strategies, such as this EDS, to provide safety and efficacy information to guide the rapid and wide-scale transition to new ARV drugs.

## Supporting information

S1 File Adverse event screening form. (PDF)

S2 File Additional information on the EDS. (DOCX)
