# Supplementary material for: Monitoring the transition to new antiretroviral treatment regimens through an enhanced data system in Kenya
Source: PLoS One. 2020 Apr 23;15(4):e0232104. doi: 10.1371/journal.pone.0232104 (PMC7179904; doi:10.1371/journal.pone.0232104)
Supplement: S1 File — (PDF) [file pone.0232104.s001.pdf]

ADVERSE EVENTS / ADVERSE DRUG REACTIONS SCREENING FORM

Complete this form for PLHIV on ARV at every clinical visit

Visit Date (dd/mm/yyyy): _______________

**Patient Details:**		
Patient Name: _______________	Unique Patient Number: ________________	
Patient DoB: _______________	Sex: M __________ F __________	
**Pregnancy Status and Contraception Use:**		
Female LMP (dd/mm/yyyy): ________________________	Pregnant: Yes _____ No _____ Not sure _____	If pregnant,<br>Gestation in weeks: ____________<br>EDD (dd/mm/yyyy): _______________

If not pregnant, are you on any contraceptives?

No ____

If no, indicate whether:

i. Contraception method initiated (table below)

ii. Intention to become pregnant

iii. Positive pregnancy test

Yes ____

(If yes, indicate the contraceptive method in use below)

Contraceptive method in use	Initiation / Date<br>(tick and add date - dd/mm/yyyy)	Continuation/Date<br>(tick and add date - dd/mm/yyyy)
IM medroxyprogesterone (DMPA; Depo Provera)		
Norethisterone enanthate (NET-EN; norethindrone)		
Implants		
Combined oral contraceptive (pill)		
Intrauterine device (IUD)		

Condoms		
Emergency contraceptive pill (ECP)		
Sterilization		
Fertility awareness-based (FAB) methods		
Lactational amenorrhoea method (LAM)		
Spermicides and diaphragm		
**ARV Regimen Data:**		
ART History		
ART start date (dd/mm/yyyy): ________________<br>All regimen substitutions and switches including dates since ART initiation:		
1st line:	2nd line:	3rd line:
Reason for substitution of ART regimen<br>☐ ARV substitution from legacy 1st line regimen<br>☐ Substitution for NRTIs<br>☐ Substitution for NNRTI<br>☐ Substitution for PI/r in PWID<br>☐ Substitution due to EFV intolerance / toxicity<br>☐ Other (specify):	Reason for substitution of ART regimen<br>☐ ARV substitution from PI/r to DTG due to TB / HIV co-infection in 2nd line regimen<br>☐ Substitution due to intolerance / toxicity<br>☐ Other (specify):	Reason for substitution of ART regimen<br>☐ ARV substitution from RAL in 3rd line regimen<br>☐ Substitution due to intolerance / toxicity<br>☐ Other (specify):
1st line:	2nd line:	3rd line:
Reason for substitution of ART regimen<br>☐ ARV substitution from legacy 1st line regimen<br>☐ Substitution for NRTIs<br>☐ Substitution for NNRTI<br>☐ Substitution for PI/r in PWID	Reason for substitution of ART regimen<br>☐ ARV substitution from PI/r to DTG due to TB / HIV co-infection in 2nd line regimen<br>☐ Substitution due to intolerance / toxicity<br>☐ Other (specify):	Reason for substitution of ART regimen<br>☐ ARV substitution from RAL in 3rd line regimen<br>☐ Substitution due to intolerance / toxicity<br>☐ Other (specify):

☐ Substitution due to EFV intolerance / toxicity<br>☐ Other (specify):		
Current Medications/ Treatment		
ARV regimen and dose:	Any OI prophylaxis or treatment and dose:	Other treatment including dose:

Symptom Screening		
**Assess for the following**	**Indicate Yes or No**	**Severity of the symptom. Indicate as:**<br>□ Mild (Grade 1)<br>□ Moderate (Grade 2)<br>□ Severe (Grade 3)<br>□ Life threatening (Grade 4)
Abdominal discomfort/abdominal pain		
Abnormal dreams or nightmares (Frightening or unpleasant dreams)		
Anaemia		
Anxiety (nervousness, fear, apprehension, and worrying)		
Burning sensation and tingling in limbs/ Paresthesia/painful neuropathy		
Confusion/abnormal thinking		
Depression/mood changes (frequently feeling very low)		
Diarrhoea		
Dizziness/spinning sensation/vertigo		
Fat changes/lipodystrophy		
Fatigue/tiredness/weakness		
Gynaecomastia		
Headache		

Insomnia (lacking sleep at night)/sleep problems		
Jaundice		
Nausea		
Pancytopenia		
Poor concentration/ memory problems		
Renal failure/renal insufficiency		
Suicide ideation (thoughts on ending the life)		
Skin rash/hypersensitivity reaction		
Vomiting		
Other Specify:		

**If <u>Yes</u> to any symptomatology above assess for adherence to ART, immediate action taken, and patient outcome**	
**Adherence to ART:**<br>Has patient skipped a dose of ARV due to these symptoms? (Indicate Yes or No) ___________<br>If Yes indicate number of missed doses: ______________	
**Immediate Action Taken**:<br>□ Regimen changed<br>□ Regimen not changed<br>□ Regimen stopped	**Other actions taken**<br>□ Laboratory investigations requested, Specify:<br>____<br>□ Other, Specify: _____________________________________

**If <u>Yes</u> to any symptomatology above assess for the resolution of the AE at subsequent clinical visits**						
Patient Outcome after ADR:	Visit 1<br>dd/mm/yyyy<br>_____________	Visit 2<br>dd/mm/yyyy<br>_____________	Visit 3<br>dd/mm/yyyy<br>_____________	Visit 4<br>dd/mm/yyyy<br>_____________	Visit 5<br>dd/mm/yyyy<br>_____________	Visit 6<br>dd/mm/yyyy<br>_____________
Recovering / resolving						
Recovered / resolved						

Requires or prolongs hospitalization						
Requires intervention to prevent permanent damage						
Died, due to ADR						
Died, not due to ADR						
