# Supplementary material for: Monitoring the transition to new antiretroviral treatment regimens through an enhanced data system in Kenya
Source: PLoS One. 2020 Apr 23;15(4):e0232104. doi: 10.1371/journal.pone.0232104 (PMC7179904; doi:10.1371/journal.pone.0232104)
Supplement: S2 File — (DOCX) [file pone.0232104.s002.docx]

**Supplemental File 2.** Additional information on the de-duplication of the EDS

We developed a patient matching algorithm to support de-duplication of patient records at facility level and to prevent multiple registrations of the same client. DWAPI uses a combination of both deterministic and probabilistic matching of the patient demographics.

**Summary of approaches to deduplication/matching**

1. **Soundex:**

Applied on the first name (Usually better for English Names)

1. **Double metaphone:**

Applied on the second name (Usually better for African Names)

1. **Output from 1 & 2:**

Concatenated with gender and date of birth (DoB) to generate a PKV (Patient Key Value)

1. **Jaro-winkler (Probabilistic scoring):**

Uses the PKV to compute a matching score against other PKV and generate a list of possible duplicates. The score is used to set a threshold for automated/manual merging of patient records

1. **Deterministic matching**

Using other MPI variables to double check & verify the matches generated from the above approach

**Preparing the MPI variables for matching:**

The MPI data is prepared for parsing as follow;

Step 1: All names (patient Name and Nok Names) are normalized and stripped off leading and trailing spaces, comma and any special characters

Step 2: All dates are converted to yyyymmddd format

Step 3: Sex variable is mapped to M, F

After data is normalized and prepared for deduplication a **Patient Key Variable (PKV)** is created by:

1. Prefix Sex of patient
2. Concatenate with the Soundex values of FirstName
3. Concatenating with the double metaphone of the LastName
4. Concatenate with the DOB in ISO format (yyyymmdd)

The resulting PKV is <gender>soundex(firstname)dm(lastName)DoB.

The deduplication algorithm starts with a deterministic pass through the data as described as in the flow below;

*Deterministic Matching*

Deterministic matching is carried out in two phases as outlined below.

Phase One: Matching CCC Numbers

1. Step 1: Group the data set on CCC Numbers to identify any duplicate CCC numbers. Assumption: CCC number is a Unique number for the HIV program.
2. Step 2: For each record found in a group, compare the **Patient Key Variable** **(PKV) – the output of this PKV is stored in a database for purposes of faster querying of the duplicate records.**
3. Step 3: If PKV matches, this is a duplicate record go to step 4 else go to step 5
4. Step 4: Merge the patient profile and delete one record (Log delete in Audit table with reason for delete as “duplicate”)
5. Step 5: PKV does not match do nothing to records.
6. Step 6: Move to next group – See step 2

**Outcome**: All duplicate CCC numbers that have 100% match on PKV are collapsed into one record and duplicate record is flagged.

Phase Two: Matching PKV

Assumptions: (1) Soundex algorithm detects spelling variations in names that sound the same. (2) double metaphone algorithm detects spelling variations on African Names (3) Phase one flagged all possible CCC Number duplicates.

1. Step 1: Group the data set on PKV to find any duplicate PKV records.
2. Step 2: For each record found in a group compare the patient-telephone-numbers. If matched, go to 3 else go to 4
3. Step 3: For matches flag as possible duplicate, Generate Patient UPI Key and link the UPI key and CCC Numbers in the **Patient Program Numbers** table. Go to 7
4. Step 4: If not matched, compare Patient-NOK-telephone-Numbers, if matched go to 3 else go to 5
5. Step 5: If NOK details not matched, compare Patient Start ART Date & Regimens details if matched go to 3 else go to 6
6. Step 6: If not matched, flag record for probabilistic matching using different algorithm such as Jaro-Winkler.
7. Step 7: Move to next group – See step 2

*Probabilistic Matching*

For probabilistic matching, the project uses the PKV and applies the Jaro-Winkler distance algorithm to detect possible duplicate patients. A threshold of 0.96 based is applied currently. For records identified as possible matches they are then applied to the algorithm below:

1. Step 1: Group data by PKV values and Jaro-winkler score.
2. Step 2: For each record found in a group compare the patient-telephone-numbers. If matched, go to 3 else go to 4
3. Step 3: For matches flag as possible duplicate, Generate Patient UPI Key and link the UPI key and CCC Numbers in the **Patient Program Numbers** table. Go to 7
4. Step 4: If not matched, compare Patient-NOK-telephone-Numbers, if matched go to 3 else go to 5
5. Step 5: If NOK details not matched, compare Patient Start ART Date & Regimens details if matched go to 3 else go to 6
6. Step 7: Move to next group – See step 2
